# Light-Induced ^1^H NMR Hyperpolarization in Solids at 9.4 and 21.1 T

**DOI:** 10.1021/jacs.4c06151

**Published:** 2024-07-15

**Authors:** Federico De Biasi, Ganesan Karthikeyan, Máté Visegrádi, Marcel Levien, Michael A. Hope, Paige J. Brown, Michael R. Wasielewski, Olivier Ouari, Lyndon Emsley

**Affiliations:** † Institut des Sciences et Ingenierie Chimiques, École Polytechnique Fedérale de Lausanne (EPFL), CH-1015 Lausanne, Switzerland; ‡ Aix-Marseille University, CNRS, Institut de Chimie Radicalaire, 13013 Marseille, France; § Department of Chemistry, Center for Molecular Quantum Transduction, Paula M. Trienens Institute for Sustainability and Energy, Northwestern University, Evanston, Illinois 60208-3113, United States

## Abstract

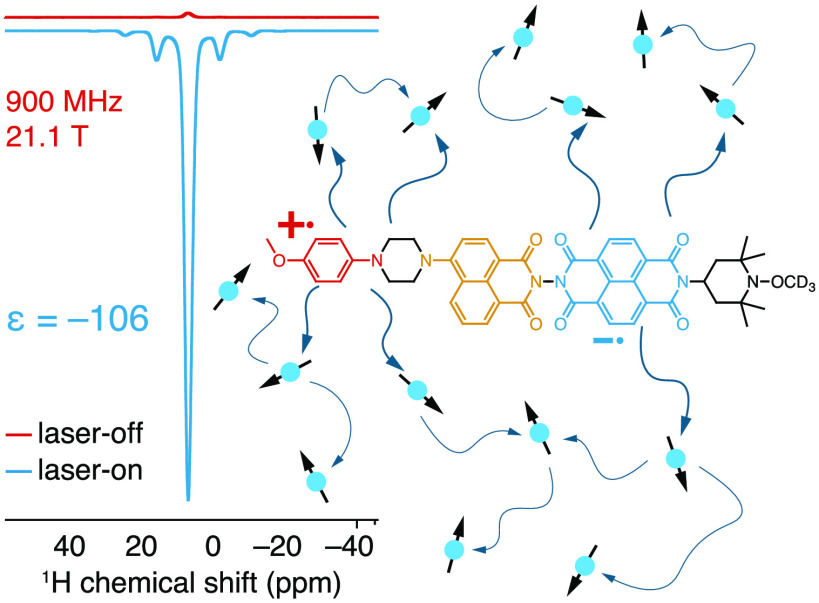

The inherently low sensitivity of nuclear magnetic resonance (NMR) spectroscopy is the major limiting factor for its application to elucidate structure and dynamics in solids. In the solid state, nuclear spin hyperpolarization methods based on microwave-induced dynamic nuclear polarization (DNP) provide a versatile platform to enhance the bulk NMR signal of many different sample formulations, leading to significant sensitivity improvements. Here we show that ^1^H NMR hyperpolarization can also be generated in solids at high magnetic fields by optical irradiation of the sample. We achieved this by exploiting a donor–chromophore–acceptor molecule with an excited state electron–electron interaction similar to the nuclear Larmor frequency, enabling solid-state ^1^H photochemically induced DNP (photo-CIDNP) at high magnetic fields. Through hyperpolarization relay, we obtained bulk NMR signal enhancements ε_H_ by factors of ∼100 at both 9.4 and 21.1 T for the ^1^H signal of *o*-terphenyl in magic angle spinning (MAS) NMR experiments at 100 K. These findings open a pathway toward a general light-induced hyperpolarization approach for dye-sensitized high-field NMR in solids.

Nuclear magnetic resonance (NMR) spectroscopy is currently one of the most versatile probes of structure and dynamics in solids.^1^ However, the intrinsically low sensitivity of NMR constitutes a bottleneck to its application in many systems. As a result, techniques to provide nuclear spin hyperpolarization have become of central importance, in both the solid and solution states.^2−10^

For solids, with the advent of high-frequency high-power microwave sources suitable for NMR experiments at 5 T and above,^11,12^ microwave-induced dynamic nuclear polarization (DNP)^13,14^ has developed into the most general method to generate hyperpolarization.^12,15,16^ Today, in a typical microwave-induced DNP experiment, a material under investigation is coformulated with a polarizing agent (PA) that acts as a source of electron spin polarization. The sample is cooled to cryogenic temperatures and, upon microwave irradiation, the large thermal electron spin polarization is transferred from the PA to nearby ^1^H nuclei and successively, by spontaneous ^1^H–^1^H spin diffusion, to the entire nuclear spin network.^17−20^ This strategy has the advantage that the material under investigation does not have to be DNP-active itself,^21^ thereby enabling sensitivity-enhanced solid-state NMR of a very wide range of materials.

Hyperpolarization in solids can also be generated optically.^22−29^ Optical methods exploit transient electronic excited states as a source for nuclear spin hyperpolarization, and they can lead to very high transient spin polarization levels,^26,30−34^ in principle up to unity. Photochemically induced dynamic nuclear polarization (photo-CIDNP) is an NMR hyperpolarization technique that only requires light irradiation of the sample,^35−37^ and which can be active in solids.^38−40^ In solid-state photo-CIDNP, light is absorbed by a donor–acceptor system whose excited state undergoes charge separation, generating a transient spin-correlated radical pair (SCRP).^40^ The SCRP generally consists of a cationic radical center on the donor site coupled to an anionic radical center on the acceptor site. The evolution and recombination of the SCRP can cause nuclear spin hyperpolarization to accumulate on nuclei that share a hyperfine coupling with at least one of the two radical centers, i.e., locally within the donor–acceptor system.^41,42^

Solid-state photo-CIDNP at magnetic fields >3 T has been previously observed in flavoproteins and photosynthetic reaction centers, where the effect was used mainly to determine the electronic properties of the donor–acceptor systems present within the biomolecules themselves. In these systems, only ^13^C and ^15^N photo-CIDNP activity was observed.^38,40,43−57^ The local nature of the photo-CIDNP effect, paired with the poor spin diffusion efficiency of ^13^C and ^15^N, was perfectly suited to the aim of those investigations, as only the resonances within the donor–acceptor photoactive centers were enhanced, allowing targeted detection of the spin system of interest.

We propose that if suitable synthetic molecular ^1^H photo-CIDNP polarizing agents can be developed, they would enable a novel hyperpolarization protocol for dye-sensitized solid-state NMR, analogous to microwave-induced magic angle spinning (MAS) DNP, where the polarization is first generated locally (within or nearby the PA) by irradiation of the sample with light, and then relayed to the bulk via spin diffusion.

Toward this end, we previously reported the first example of ^1^H photo-CIDNP in solids by optical irradiation of a synthetic donor–chromophore–acceptor (D–C–A) system, dubbed PhotoPol (Figure 1) at 0.3 T (12.8 MHz ^1^H Larmor frequency) and 85 K.^58^ We also demonstrated that spontaneous ^1^H–^1^H spin diffusion enabled relay of the polarization from the PhotoPol polarizing agent to the bulk, leading to uniform ^1^H hyperpolarization of the frozen glassy matrix in which PhotoPol was dissolved. Nevertheless, photo-CIDNP is expected to be magnetic field dependent,^46,47,59−61^ and PhotoPol yields only minor ^1^H photo-CIDNP activity at high magnetic fields (e.g., 400 MHz, Figure S2).

**Figure 1:**
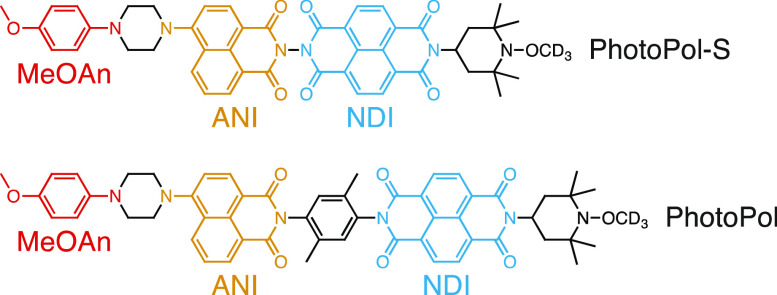
Structures of PhotoPol-S and PhotoPol. The donor (MeOAn), chromophore (ANI) and acceptor (NDI) are shown in red, yellow and blue, respectively. The linkers and end group are shown in black.

Here we report a second molecular D–C–A ^1^H photo-CIDNP polarizing agent, dubbed PhotoPol-S (Figure 1), designed to operate at high NMR frequencies. The PA is tailored to have an excited state electron–electron interaction similar to the ^1^H Larmor frequency at typical NMR magnetic fields, and we demonstrate ^1^H NMR signal enhancement factors, ε_H_, of ∼100 at both 400 and 900 MHz for *o*-terphenyl in dye-sensitized MAS NMR experiments at 100 K.

In theory, the three-spin mixing (TSM)^60,61^ photo-CIDNP mechanism is active if the magnitude of the electron–electron interaction in the excited state SCRP, *d* = −*D* – 2*J*_ex_, matches the nuclear Larmor frequency (ω_N_).^41,42,62^ The measured value for *d* in PhotoPol is −5.5 MHz (at 85 K in toluene).^30^ The spin-correlated radical centers are located on the donor and the acceptor, and it is well established that shortening the donor–acceptor distance in D–C–A molecules enhances both the SCRP electron–electron dipolar interaction (*D*) and the electron–electron exchange interaction (2*J*_ex_).^30,63−69^ In this light, we recently introduced CarboPol, a synthetic D–C–A system that shows strong ^13^C photo-CIDNP activity at 100.6 MHz (9.4 T), and which has a *d* of approximatively −56 MHz (at 85 K in butyronitrile).^70^

Following the same design principle, here we report PhotoPol-S, whose D–C–A structure is similar to that of PhotoPol (Figure 1). In both molecules, D is 4-methoxyaniline (MeOAn), C is 4-aminonaphthalene-1,8-dicarboximide (ANI) and A is naphthalene-1,8:4,5-bis(dicarboximide) (NDI). The only difference between the two polarizing agents is the absence of the xylyl spacer in PhotoPol-S between ANI and NDI, reducing the donor–acceptor distance and increasing the SCRP electron–electron interaction. Indeed, 2*J*_ex_ increases from 14 MHz (PhotoPol) to 560 MHz (PhotoPol-S), as measured at 85 K. Simultaneously, the dipolar interaction (*D*) is expected to change from −8.5 to −17 MHz (based on the donor–acceptor distance).^30,63^ Further details are given in the Supporting Information (SI).

PhotoPol-S was synthesized as detailed in the SI. 20 μL of a 1.5 mM solution of PhotoPol-S in *o*-terphenyl-*d*_14_ (OTP-*d*_14_, Cambridge Isotopes) was packed into a 3.2 mm sapphire rotor to allow for light irradiation. OTP was chosen as the glassy matrix because of its solvation and glass forming properties, optical transparency, and long nuclear spin *T*_1_.^71−74^ The solution was confined to the center third of the rotor using a PTFE insert. ^1^H photo-CIDNP NMR experiments were performed at 9.4 and 21.1 T, corresponding to 400 and 900 MHz ^1^H Larmor frequencies, using Bruker NMR spectrometers (Avance III HD at 9.4 T and Avance Neo at 21.1 T) and commercial low temperature 3.2 mm MAS DNP probes. Sample irradiation was achieved with a continuous wave 450 nm blue laser coupled to an optical fiber with a bare end (flat cleave). To achieve maximum sample irradiation, the bare end of the fiber was passed through the microwave waveguide of the DNP probe and placed as close to the NMR coil as possible. The laser output power at the bare end was between 1.2 and 1.3 W. See the SI for further details. Laser-on experiments were performed with continuous irradiation. Prior to the NMR experiments, the sample was degassed by means of three freeze–pump–thaw cycles to reduce the O_2_ content dissolved in the glassy matrix.

The residual protonation in OTP-*d*_14_ was measured via solution-state NMR and mass spectrometry to be 1.5%, corresponding to ∼1 M ^1^H concentration in the OTP matrix (Figure S4). We will refer to this matrix as 98.5% OTP-*d*_14_ hereafter.

Figure 2 shows the ^1^H NMR spectra obtained at 400 and 900 MHz with and without light irradiation for a 1.5 mM frozen solution of PhotoPol-S in 98.5% OTP-*d*_14_ recorded at ∼100 K. All spectra were acquired with 8 kHz MAS and a 70 s recycle delay. The observed signal corresponds to the residual ^1^H spins in the frozen OTP matrix ([^1^H] ≈ 1 M), which are hyperpolarized by spontaneous ^1^H–^1^H spin diffusion from the PhotoPol-S source molecules. The measured signal enhancements are ε_1H_ = −106 ± 1 at 900 MHz and −88 ± 1 at 400 MHz (ε_1H_ = *I*_on_/*I*_off_ with *I*_on_ and *I*_off_ being the integrated signal intensities in the laser-on and laser-off spectra).

**Figure 2:**
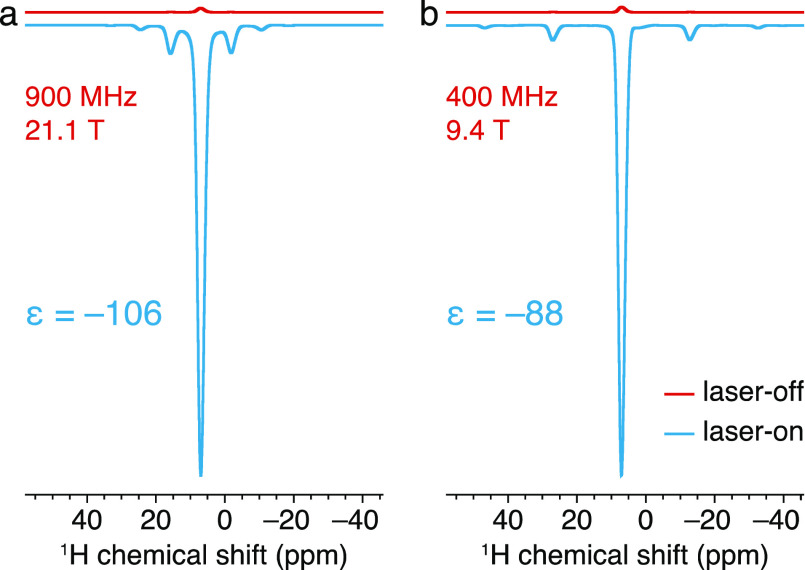
^1^H NMR spectra of a frozen solution of 1.5 mM PhotoPol-S in 98.5% OTP-*d*_14_ recorded at (a) 900 MHz and (b) 400 MHz, with and without continuous 450 nm laser irradiation (blue and red traces, respectively). All spectra were detected with a 70 s recycle delay and 8 kHz MAS using a Hahn echo block (4 rotor periods per half echo delay) to suppress the probe background, and 4 scans per experiment.

The polarization buildup with and without light irradiation were measured at both fields (Figure 3). The pulse sequence used for the experiments is outlined in Figure S5. At both fields, the buildup of bulk ^1^H hyperpolarization in the presence of light is faster than *T*_1_ (*T*_1_ > *T*_b_, Figure 3a and b), therefore the enhancement decreases with increasing polarization delay (Figure 3c and d). This is in line with a situation where the local photo-CIDNP on the PA builds up faster than its *T*_1_,^75^ and where polarization diffuses from the PA to the bulk.^17−20,76−78^ From the bulk ^1^H concentration and the ^1^H longitudinal relaxation time, the ^1^H–^1^H spin diffusion length in OTP at both fields is estimated to be >200 nm,^19,58,79,80^ much larger than the average distance between neighboring PhotoPol-S molecules at 1.5 mM (∼13 nm, calculated as twice the Wigner-Seitz radius). We therefore expect the OTP matrix to be uniformly polarized at all the polarization delays studied here. Moreover, increasing the overall matrix protonation to 5%, by the addition of protonated OTP, causes |ε_1H_| to decrease from ∼100 to ∼60, as measured at 900 MHz (Figure S3). These observations suggest that spin diffusion between the bulk nuclei is not the limiting factor for the development of bulk nuclear hyperpolarization.^20,76,77^

**Figure 3:**
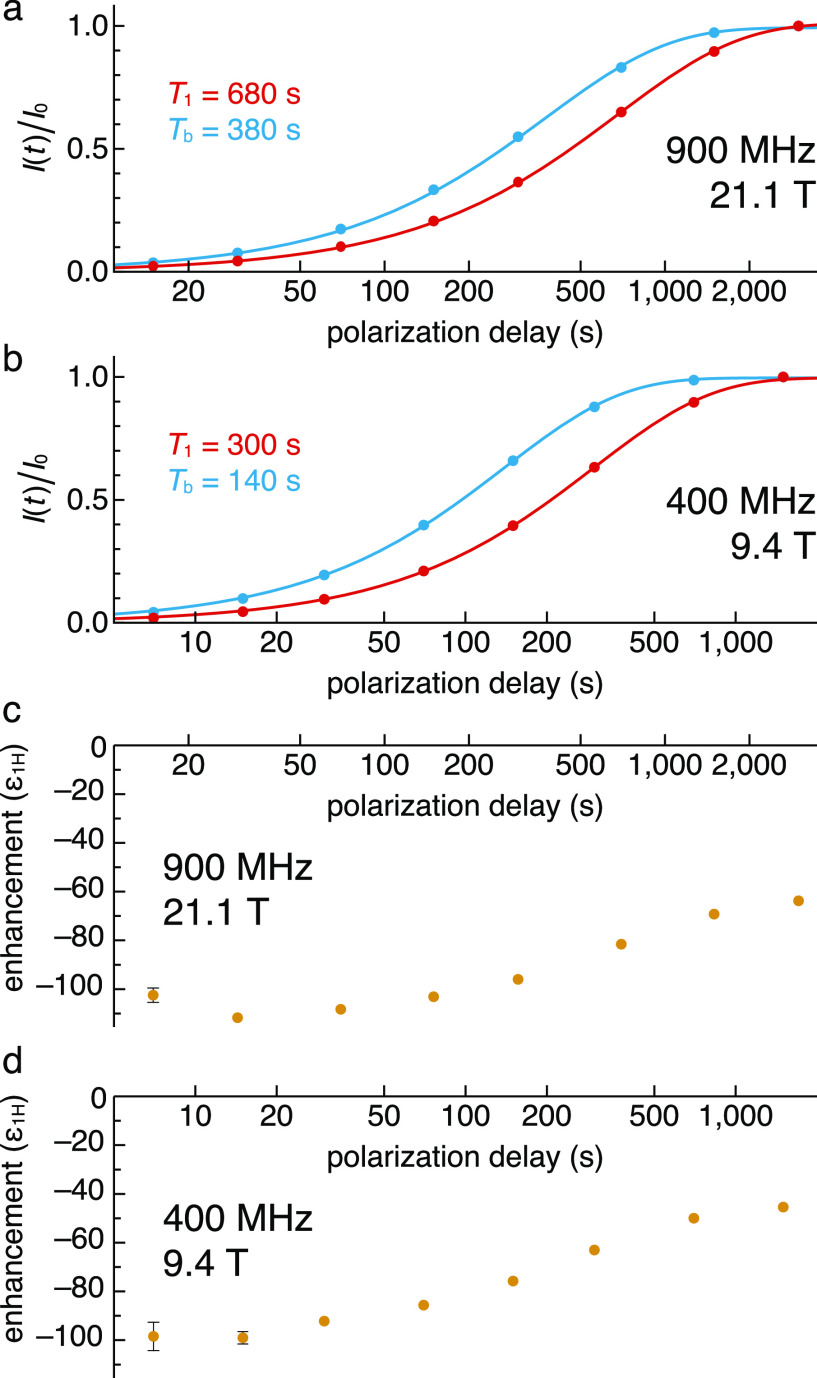
(a, b) Polarization buildup and longitudinal relaxation of the ^1^H NMR signal from a 1.5 mM frozen solution of PhotoPol-S in 98.5% OTP-*d*_14_ measured at (a) 900 MHz and (b) 400 MHz in the absence (*T*_1_, red) and in the presence (*T*_b_, blue) of continuous 450 nm laser irradiation. The solid lines show best fits to an exponential recovery function *I*(*t*) = *I*_0_(1 – e^*–t*/*T*^), that yield the time constants indicated in the inset. (c, d) Signal enhancement as a function of the polarization delay, ε_1H_ = *I*_on_/*I*_off_, measured at (c) 900 MHz and (d) 400 MHz. Error bars that are smaller than the symbol size have been omitted.

Based on the large electron–electron coupling designed for PhotoPol-S, the active ^1^H photo-CIDNP mechanism is expected to be TSM. Indeed, TSM can occur when |ω_N_| ≈ |*d*|, with the caveat that *d* is much larger than any of the electron–nuclear hyperfine couplings.^41,42,62^^1^H hyperfine couplings in PhotoPol and PhotoPol-S are expected be less than 50 MHz,^30^ hence the condition holds for PhotoPol-S. The TSM matching condition is not perfectly satisfied for ^1^H spins at either of the two explored fields, as *d* ≈ −560 MHz for most orientations of the PhotoPol-S molecule. Nevertheless, the TSM condition is expected to be sufficiently broad to be active at both fields, although with reduced efficiency.

In conclusion, we have reported the first examples of optically induced bulk ^1^H hyperpolarization in spinning solids via photo-CIDNP at high magnetic fields, here 9.4 and 21.1 T. The enhancements are obtained using a molecular photo-CIDNP polarizing agent with a D–C–A framework and an electron–electron interaction tailored to enable ^1^H three-spin mixing at high magnetic field. The NMR signal enhancement at both fields is on the order of a hundred fold, clearly demonstrating the efficiency of hyperpolarization relay from PhotoPol-S to the sample bulk, even at relatively low concentration of the polarizing agent (1.5 mM).

We speculate that the long hyperpolarization buildup time with light illumination might be due to the slow rate at which ^1^H nuclear hyperpolarization is developed within the PhotoPol-S molecules themselves, as suggested by previous observations of local ^13^C photo-CIDNP buildup in a similar system.^70^ We envision that the hyperpolarization efficiency can be improved by optimizing the structure of the polarizing agent, potentially enabling larger signal enhancements and faster buildup of relayed hyperpolarization. The findings open a pathway toward a general light-induced hyperpolarization approach for dye-sensitized high-field NMR in solids.

## Data Availability

All the raw NMR data associated with the manuscript can be accessed at the following link https://doi.org/10.5281/zenodo.12685639 and is available under the CC-BY-4.0 (Creative Commons Attribution-ShareAlike 4.0 International) license.

## Supporting Information Available

Synthetic methods and characterization of PhotoPol-S, details on the probe modifications and light source used here, supplementary figures, and a link to all the raw NMR data (PDF)
