# Supplementary material for: Light-Induced 1H NMR Hyperpolarization in Solids at 9.4 and 21.1 T
Source: J Am Chem Soc. 2024 Jul 15;146(29):19667–72. doi: 10.1021/jacs.4c06151 (PMC11273353; doi:10.1021/jacs.4c06151)
Supplement: Supplementary file 1 — ja4c06151_si_001.pdf [file ja4c06151_si_001.pdf]

Light-induced ^{1}H NMR hyperpolarization in solids at 9.4 and 21.1 T

Federico De Biasi,[1] Ganesan Karthikeyan,[2] Máté Visegrádi,[1] Marcel Levien,[1] Michael A. Hope,[1,‡] Paige J. Brown,[3] Michael R. Wasielewski,[3] Olivier Ouari,[2] and Lyndon Emsley[1,*]

[1] *Institut des Sciences et Ingenierie Chimiques, École Polytechnique Fedérale de Lausanne (EPFL), CH-1015 Lausanne, Switzerland*

[2] *Aix-Marseille University, CNRS, Institut de Chimie Radicalaire, 13013 Marseille, France*

[3] *Department of Chemistry, Center for Molecular Quantum Transduction, Paula M. Trienens Institute for Sustainability and Energy, Northwestern University, Evanston, IL 60208-3113, USA*

Raw NMR data

All the raw NMR data associated with the manuscript can be accessed at the following link DOI: 10.5281/zenodo.12685639 and is available under the CC-BY-4.0 (Creative Commons Attribution-ShareAlike 4.0 International) license.

Summary

1) Light irradiation under magic angle spinning....S2

2) Additional NMR experiments....S3

3) Electron–electron interaction in PhotoPol-S....S4

4) Pulse sequence for polarization buildup measurement....S4

5) Synthetic procdure....S4

6) References....S6

1) Light irradiation under magic angle spinning

Sample irradiation under MAS was achieved by coupling a 450 nm blue laser to an optical fiber with a bare end (ThorLabs M137L03). The fiber was guided through the empty microwave waveguide of a commercial Bruker 3.2 mm MAS DNP probe, placing the bare end as close to the NMR coil as possible. The fiber is flexible enough to pass through the double miter bend of the waveguide. Fig. S1 shows the end result achieved on a 900 MHz 3.2 mm MAS DNP probe.

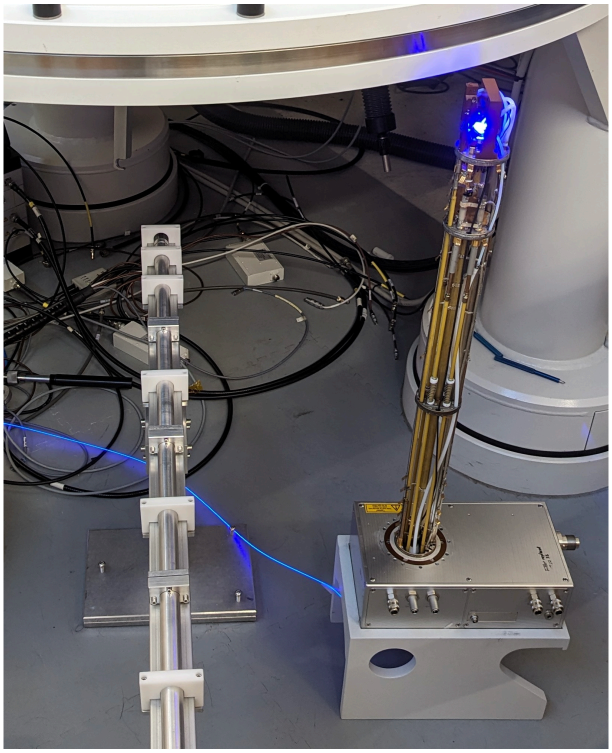

Figure S1. Picture of a Bruker 900 MHz 3.2 MAS DNP probe (without its sheath) that was used for the ^{1}H photo-CIDNP experiments at 21.1 T. Light was delivered to the stator through an optical fiber placed inside the empty microwave waveguide. The picture was taken with the laser on at maximum power.

At both fields, the sample was spun with a Bruker MAS II unit. It has been found that, without any sample in the stator, the 450 nm laser light triggered the MAS reading, reaching a value of roughly 90,000 Hz at maximum laser power with 600 mbar of both cold bearing and drive gas and 2000 lph VT gas flow. During this test, the cold gas flows were applied only to maintain the probe at low T and did not actually spin the sample. In the presence of a spinning sample, the automatic MAS rate stabilization was disabled when powering the laser on and during the entire duration of the laser-on experiments to avoid any interference with the MAS reading. Without automatic stabilization, the MAS rate in the experiments was 8000 ± 20 Hz.

The blue laser used to irradiate the sample consists in a 450 nm diode (3000 mW nominal output power) with adjustable focal length controlled by an ACAN A12 driver. The laser is coupled to a λ/2 waveplate and a polarizing beam splitter to adjust the output power. Maximum power was used in all the NMR experiments, resulting in ~1.2 W output power at the bare end of the optical fiber.

2) Additional NMR experiments

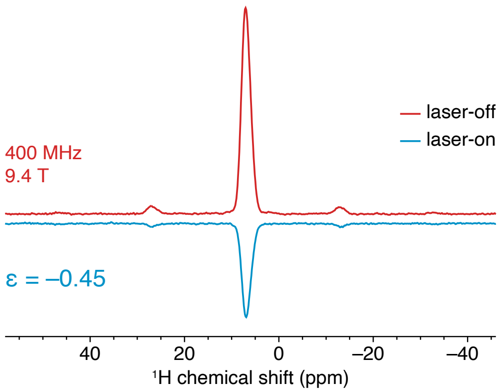

Figure S2. ^{1}H NMR spectra of a 1.5 mM PhotoPol frozen solution in 98.5% OTP-d_{14} recorded at 400 MHz, with and without continuous 450 nm laser irradiation (blue and red traces, respectively). Spectra were detected with a 70 s recycle delay and 8 kHz MAS using a Hahn echo block (4 rotor periods per half echo delay) to suppress the probe background, and 4 scans per experiment.

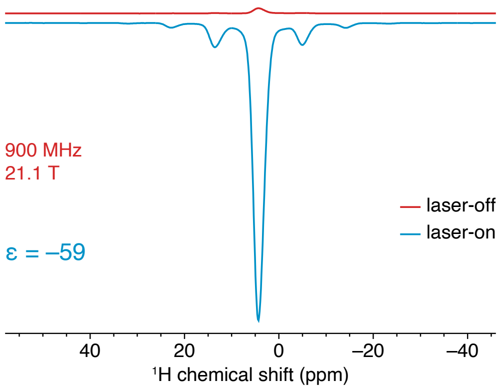

Figure S3. ^{1}H NMR spectra of a frozen solution of 1.5 mM PhotoPol-S in OTP-d_{14} (with the overall protonation level of the OTP matrix was 5%, obtained by mixing OTP-d_{14} (98.5 %) with fully protonated OTP) recorded at 900 MHz, with and without continuous 450 nm laser irradiation (blue and red traces, respectively). Spectra were detected with a 100 s recycle delay and 8 kHz MAS using a Hahn echo block (4 rotor periods per half echo delay) to suppress the probe background, and 2 scans per experiment.

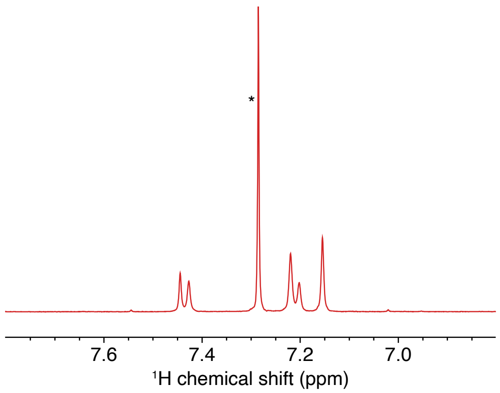

Figure S4. Solution-state ^{1}H NMR spectrum recorded at 400 MHz of a sample consisting of 490 µL $CDCl_3$, 10 µL DMSO and 16 mg OTP-d_{14} (98%, Cambridge Isotopes). Spectrum was recorded using a 200 s recycle delay (4 scans). All signals in the spectrum but that indicated by the asterisk have been assigned to OTP. The asterisk denotes the residual $CDCl_3$ signal. The ^{1}H signal of DMSO resonating at 2.6 ppm, corresponding to ^{1}H having a 1.69 M concentration, was used as an internal standard to determine the residual protonation of OTP-d_{14}. By comparison of the DMSO signal integral with the sum of integrals of the

OTP signals, the overall concentration of the OTP residual protons is calculated to be 30 mM, corresponding to a 1.5% residual protonation of OTP-d_{14}.The deuteration level was also verified by MS and an overall deuteration level of 98.25% was obtained. Indeed, according to the MS experiment this corresponds to a mixture of 81% OTP-d_{14}, 15% OTP-d_{13}, 3% OTP-d_{12}, 0.3% OTP-d_{11}, 0.6% OTP-d_{10}, 0.1% OTP-d_9 and 0.03% OTP-d_8.

3) Electron–electron interaction in PhotoPol-S

A sample of PhotoPol-S was prepared in butyronitrile with optical density (O.D.) = 0.5 – 0.7 at 415 nm for measurements at 85 K. The sample was placed in a borosilicate rectangular sample cuvette (2×4 mm, 0.5 mm wall), then subjected to three freeze–pump–thaw cycles on a vacuum line (10^{-4} Torr) and sealed with a hydrogen torch. The cuvette was placed in a nitrogen-cooled cryostat (STVP-100 Janis) positioned between the poles of a Walker Scientific HV-4W electromagnet powered by a Walker Magnion HS-735 power supply. The field strength was measured by a Lakeshore 475 DSP Gaussmeter with a Hall effect probe. The sample was pumped using 415 nm, 5 ns laser pulses at a 1 kHz repetition rate from an NT242 Ekspla laser. The white light probe pulses were generated at 2 kHz repetition rate using a Leukos white light supercontinuum laser. After passing through the sample, the probe light was filtered using a 10 nm bandpass filter centered at 500 nm and directed into a photomultiplier tube (Hamamatsu H9307-03). The signal was then monitored using a lock-in amplifier (Stanford Research Systems SR830 DSP) with a 1 kHz reference signal from the pump laser. At 85 K, the changes in the MeOAn$^{•+}$–ANI–NDI$^{•−}$ transient absorption were monitored at a 10 µs pump–probe delay, while the magnetic field was swept from 0 to 40 mT in 0.5 mT steps. The transient absorption changes at 85 K exhibited a resonance at $B_{1/2} = 2J = 20$ mT (≈ 560 MHz).

4) Pulse sequence for polarization buildup measurement

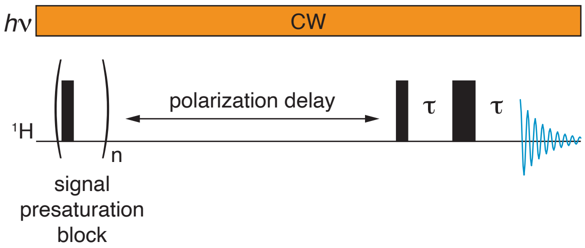

Figure S5. Pulse sequence for polarization buildup measurement used to collect the data in Fig. 3 of the main text. After presaturation of the NMR signal with a series of 90° pulses, polarization builds up during the polarization delay prior to signal acquisition. The NMR signal is acquired using a rotor-synchronized Hahn echo block to suppress the probe background signal. In the experiments, τ has been set equal to 4 rotor periods.

5) Synthetic procedure

Experimental procedures

General procedures. Unless otherwise noted, all reactions were carried out under an inert atmosphere of argon. Chemicals were used as received from the suppliers. Reactions were monitored by thin-layer chromatography (TLC) analysis. Column chromatography was carried out on silica gel (230-400 mesh). The ^{1}H NMR spectra were recorded on a Bruker AVL spectrometer at 500 MHz. The chemical shifts are reported in ppm downfield relative to TMS and referenced using the residual $CHCl_3$ resonance (δ = 7.26) for ^{1}H NMR. ESI-HRMS was performed on a SYNAPT G2 HDMS (Waters). The NMR and MS analysis were performed at the Spectropole facilities, Marseille. Compounds **A**[1] and **B**[2] were prepared as previously reported.

Synthesis of PhotoPol- S

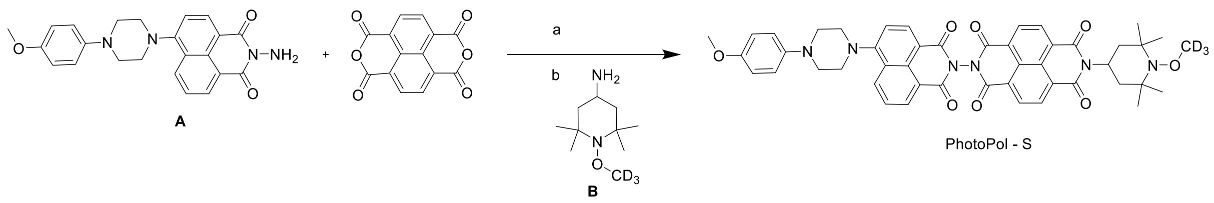

Scheme S1. Reagents and conditions: a) Pyridine, 110 °C, 6hrs. b) Compound **B**, 110 °C, 15hrs.

Compound **A**[1] (22 mg, 0.055 mmol) and 1, 4, 5, 8-naphthalenetetracarboxylic dianhydride (15 mg, 0.055 mmol) were dissolved in 3mL of pyridine and the mixture was heated at 120 °C under an argon atmosphere for 6 hours. Then the reaction mixture was cooled to 40 °C and 1-(methoxy-d_3)-4-amino-2,2,6,6-tetramethylpiperidine (compound **B**[2]) (11 mg, 0.055 mmol) in 0.1mL pyridine was added and continued heating at 120 °C for 15hrs. The reaction mixture was concentrated in vacuum and purified by column chromatography to give **PhotoPol - S** (10mg, 22%), R_f = 0.65 (5% MeOH/ CH_2Cl_2).

^{1}H NMR ($CDCl_3$, 500 MHz) δ= 1.26-1.32 (m, 12H), 1.52-1.60 (m, 4H), 2.84-2.89 (m, 1H), 3.43-3.51 (m, 8H), 3.82 (s, 3H), 6.73 (dd, J=8.7, 2.0 Hz, 2H), 6.87 (dd, J=9.0, 2.9 Hz, 2H), 7.34 (t, J=7.6, 1H), 7.77-7.81 (m,1H), 8.58-8.70 (m,3H), 8.71-8.91 (m,4H).

HRMS (ESI-TOF) m/z: $[M+H]^+$ calcd for $C_{47}H_{42}D_3N_6O_8^+$ 824.3482, found 824.3478.

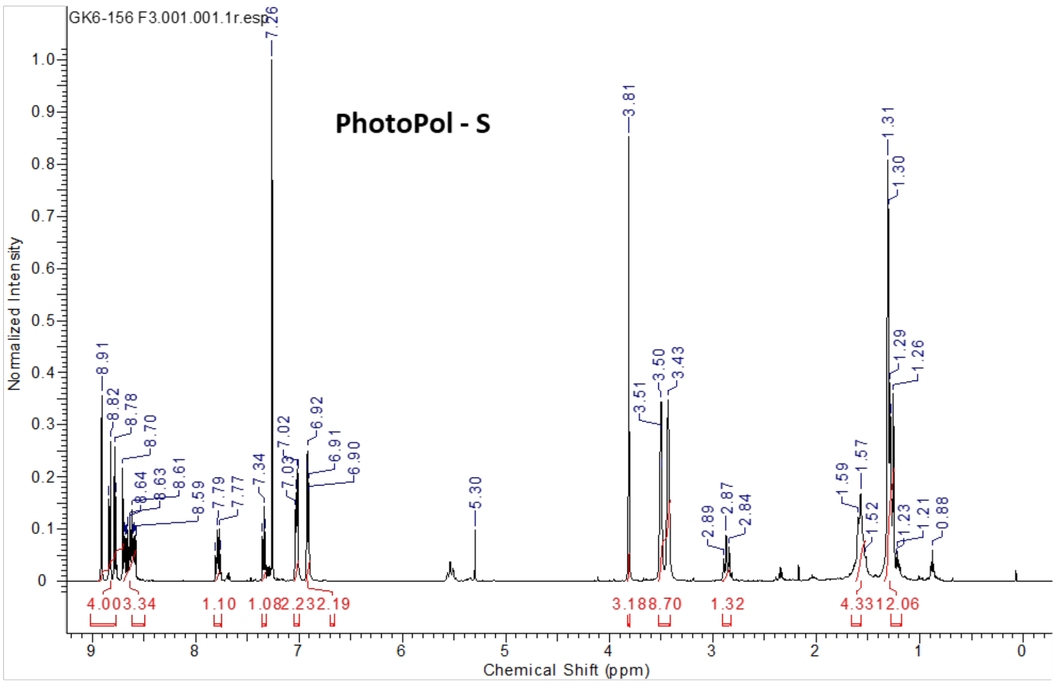

Figure S6. ^{1}H NMR spectrum (500 MHz, $CDCl_3$) of PhotoPol-S

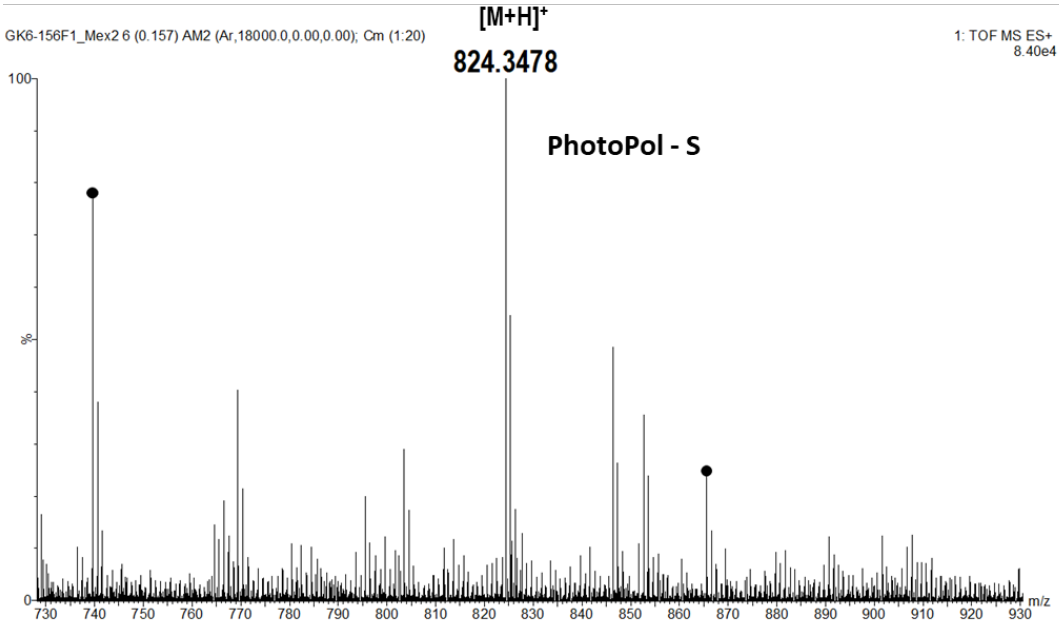

Figure S7. ESI-HRMS spectrum of PhotoPol-S (peaks indicated with a circle are due to the internal calibration).

6) References

1. Weiss, E. A.; Chernick, E. T.; Wasielewski, M. R. Modulation of Radical Ion Pair Lifetimes by the Presence of a Third Spin in Rodlike Donor-Acceptor Triads. *J. Am. Chem. Soc.* **2004**, *126*, 2326−2327.

2. De Biasi, F.; Hope, M. A.; Avalos, C. E.; Karthikeyan, G.; Casano, G.; Mishra, A.; Badoni, S.; Stevanato, G.; Kubicki, D. J.; Milani, J., *et al*., Optically Enhanced Solid-State ^{1}H NMR Spectroscopy. *J. Am. Chem. Soc.* **2023**, *145* (27), 14874-14883.
